# Reactivation of HIV-1 from Latency by an Ingenol Derivative from *Euphorbia Kansui*

**DOI:** 10.1038/s41598-017-07157-0

**Published:** 2017-08-25

**Authors:** Pengfei Wang, Panpan Lu, Xiying Qu, Yinzhong Shen, Hanxian Zeng, Xiaoli Zhu, Yuqi Zhu, Xian Li, Hao Wu, Jianqing Xu, Hongzhou Lu, Zhongjun Ma, Huanzhang Zhu

**Affiliations:** 1 State Key Laboratory of Genetic Engineering and Key Laboratory of Medical Molecular Virology of Ministry of Education/Health, Institute of Genetics, School of Life Sciences, Fudan University, Shanghai, 200438 China; 2 Department of Infectious Diseases, and Key Laboratory of Medical Molecular Virology of Ministry of Education/Health, Shanghai Public Health Clinical Center, Fudan University, Shanghai, 200433 China; 3 Center for Infectious Diseases, Beijing You’an Hospital, Capital Medical University, Beijing, 100069 China; 4 Institute of Marine Biology, Ocean College, Zhejiang University, Hangzhou, 310058 China

## Abstract

Cells harboring latent HIV-1 pose a major obstacle to eradication of the virus. The ‘shock and kill’ strategy has been broadly explored to purge the latent reservoir; however, none of the current latency-reversing agents (LRAs) can safely and effectively activate the latent virus in patients. In this study, we report an ingenol derivative called EK-16A, isolated from the traditional Chinese medicinal herb *Euphorbia kansui*, which displays great potential in reactivating latent HIV-1. A comparison of the doses used to measure the potency indicated EK-16A to be 200-fold more potent than prostratin in reactivating HIV-1 from latently infected cell lines. EK-16A also outperformed prostratin in *ex vivo* studies on cells from HIV-1-infected individuals, while maintaining minimal cytotoxicity effects on cell viability and T cell activation. Furthermore, EK-16A exhibited synergy with other LRAs in reactivating latent HIV-1. Mechanistic studies indicated EK-16A to be a PKCγ activator, which promoted both HIV-1 transcription initiation by NF-κB and elongation by P-TEFb signal pathways. Further investigations aimed to add this compound to the therapeutic arsenal for HIV-1 eradication are in the pipeline.

## Introduction

Over the past three decades, great progress has been achieved in the fight against HIV/AIDS. In adherent patients receiving combined antiretroviral therapy (cART), plasma HIV-1 levels have been suppressed to below the limit of available detection methods^1–3^. However, the virus levels soon rebound to pretreatment levels after the interruption of cART^4^. Current therapy cannot eradicate the latent HIV-1 reservoir, so the patient must maintain a lifelong treatment regimen, which causes toxic effects and considerable expense^5–7^. Hence, there is an important need to develop novel approaches that eradicate established HIV-1 infection, thereby eliminating the burden of lifelong cART.

HIV-1 latency is a key obstacle to the permanent cure of HIV-1 disease. The latently infected cells harbor integrated HIV-1 DNA in their genomes but do not generate viral particles, making them invisible to the antiviral immune response and drugs^8, 9^. Many factors are involved in the mechanisms of HIV-1 latency, including integration sites, epigenetic modifications, transcriptional and posttranscriptional regulations^9–11^. Several therapeutic approaches, involving either sterilizing cure (complete eradication of all persistent HIV-1) or functional cure (immunological control of persistent virus in the absence of therapy), are being considered to control or eliminate the HIV-1 latent reservoir^12, 13^. To eliminate the latently infected cells, researchers have proposed to reverse their latent viral state, employing compounds that interfere with the cellular mechanisms known to be associated with HIV persistence. Subsequently, the reactivated viral infected cells might be cleared via cytopathic effects, immune clearance and cell death, thereby purging the latent reservoir^12, 14^. This “shock and kill” strategy is currently considered one of the most promising strategies to accomplish an HIV-1 cure, and major research efforts are directed towards developing clinically effective latency-reversing agents (LRAs).

Several classes of LRAs have been intensively studied *in vitro* and *ex vivo*, but only a few of them have advanced to clinical trials^12, 15, 16^. The early clinical trials based on T cell activation, employing human CD3 antibodies (OKT3) and cytokines, showed modest reactivation effects but also revealed unacceptable toxicity^17, 18^. Mechanistic studies revealed that histone deacetylases (HDACs) are enzymes that contribute to the proviral gene silence and their inhibition might lead to the reactivation of the latent HIV-1^16, 19^. A number of HDAC inhibitors, such as valproic acid^20–25^, vorinostat (SAHA)^26–28^, panobinostat^29^ and romidepsin^30^, have been studied in pilot clinical trials. However, only evidence of low-level viral transcription paired with a lack of reservoir perturbation have been observed in these early eradication trials^31^. Another possible mechanism underlying latency is the insufficiency of positive transcription factors. In resting cells, transcription factors, such as NF-κB and P-TEFb, are expressed at very low levels and sequestered in the repressive forms^32^. Along this line, many classes of drugs capable of reversing HIV-1 latency are concurrently under investigation. Protein kinase C (PKC) agonists, including prostratin^33, 34^, bryostatin-1^35, 36^, and ingenol derivatives^37–39^ have been shown to reactivate HIV-1 expression by inducing of both NF-κB and P-TEFb. Bromodomain and extraterminal bromodomain inhibitors (BETis), such as JQ1^40^ and OTX015^41^ are able to reactivate HIV-1 from latency *in vitro* by P-TEFb and Tat-mediated transcriptional promotion. Many other agents with unique mechanisms, such as disulfiram^42, 43^, acitretin^44^, as well as Toll-like receptor (TLR) agonists^45–47^, have also been described for the purpose of activating the latent virus. However, in *ex vivo* experiments at clinically relevant concentrations, many of the aforementioned LRAs failed to induce HIV-1 from the latent reservoir of patients on cART^48, 49^, and their toxicity and target specificity still remain major concerns.

Natural products derived from traditional Chinese medicine provide rich resources for drug discovery, and have recently received increasing scientific attention. The discovery of artemisinin, an anti-malarial compound extracted from the traditional Chinese medicinal herb *Artemisia annua* was awarded the 2015 Nobel Prize in Physiology or Medicine^50^. Two procyanidin compounds isolated from the traditional medicinal plants *Theobroma cacao* ^51^ and *Polygonum cuspidatum* ^52^ have also been reported recently to reactivate latent HIV-1 in cell line models. Given this context, we attempted to examine active compounds from traditional Chinese medicinal herbs that may display HIV-1 latency-reversing activity. Our screening of a large library of natural compounds led to the identification of an ingenol derivative from the roots of *Euphorbia kansui* that was able to antagonize HIV-1 latency with high potency. *Euphorbia kansui* has traditionally been used for the treatment of edema, ascites, and asthma^53^. More recently, it was reported that a crude extract from *Euphorbia kansui* could reactivate latent HIV-1^54^, and a clinical trial using *Euphorbia kansui* extract powder as tea was designed for the purpose of clearing HIV-1 (clinicaltrials.gov, Identifier NCT02531295). However, *Euphorbia kansui* contains 12 ingenols, as well as many other active compounds^54, 55^. The active compound(s) among them responsible for reactivating of the virus have not been identified or characterized to date. In this study, we purify the active compounds from this medicinal herb, and demonstrate that one ingenol derivative called EK-16A has the highest potency in reversing HIV-1 latency. Our mechanistic studies indicate that it is a PKC agonist that can promote the transcription of HIV-1 by inducing both NF-κB and P-TEFb.

## Results

### Purified extracts of *Euphorbia kansui* promote HIV-1 expression

To identify natural products derived from traditional Chinese medicinal herbs that cause HIV-1 latency reactivation, we used C11 cells, a latently infected Jurkat T cell line. These cells contain a *green fluorescent protein (GFP)* gene under the control of the HIV-1 LTR, allowing to easily detect HIV-1 expression by fluorescence microscopy or flow cytometry^56, 57^. Partially purified fractions derived from a collection of over 100 traditional Chinese medicinal herbs from a repository at Zhejiang University were individually incubated with C11 cells and HIV-1 expression was monitored by percentage of GFP-positive cells using flow cytometry. We found that the fractions from the dried root of *Euphorbia kansui* that co-eluted with methylene chloride and petroleum ether could potently activate the LTR-driven GFP production (Supplementary Fig. 1).

**Figure 1:**
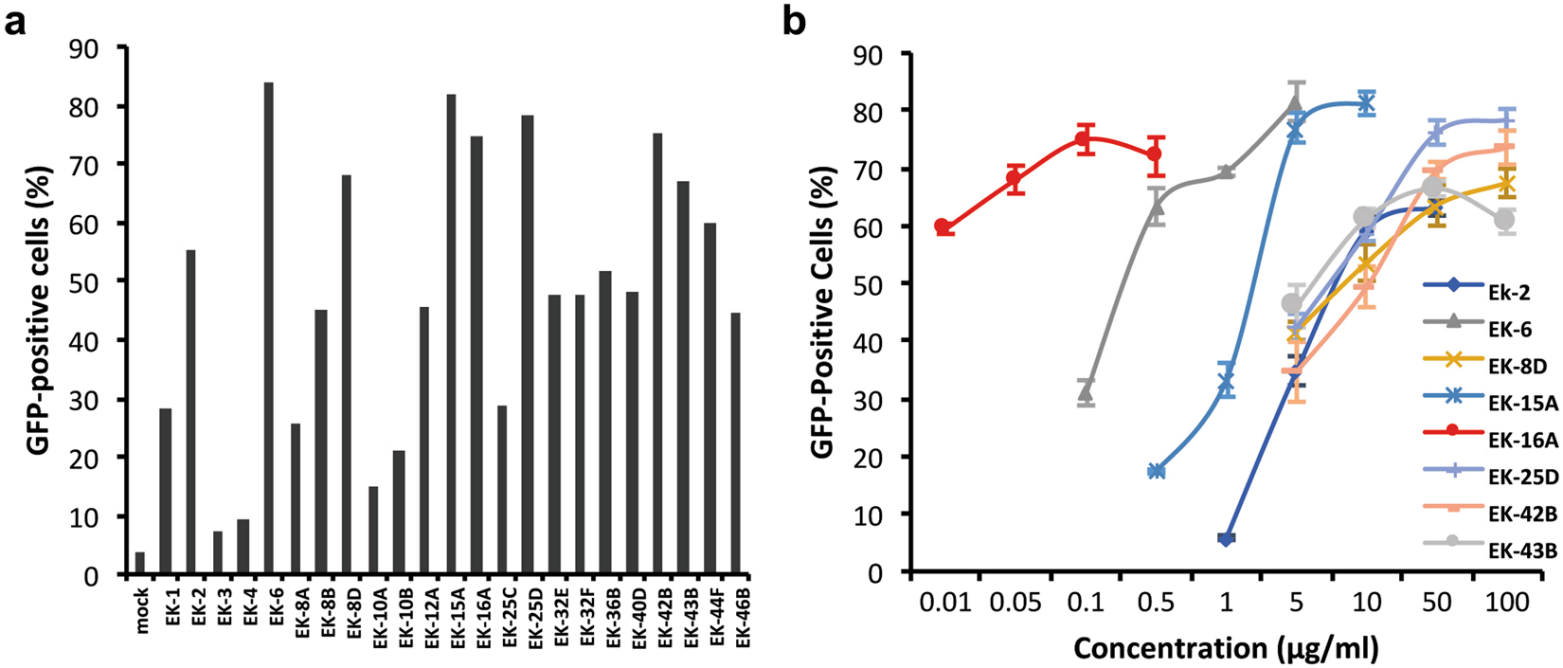
Reactivation of latent HIV-1 by compounds extracted from *Euphorbia kansui*. (**a**) C11 cells were treated with various compounds extracted from *Euphorbia kansui* at three concentrations (0.1 μg/ml, 1 μg/ml and 10 μg/ml). At 48 h post-treatment, the percentage of GFP-positive cells was measured by flow cytometry, which represented the level of HIV-1 transcription. Data show the highest GFP induction of each compound at the three concentrations tested. (**b**) C11 cells were treated with the 8 selected compounds at the indicated concentrations for 48 h and the percentage of GFP-positive cells was measured. Data show the means ± standard deviations (s.d.) of three independent experiments.

These fractions were subjected to further purification in order to identify the exact component that was responsible for HIV-1 latency reversal. More than 20 purified compounds were found to be effective at reactivating latent HIV-1, and some of the active compounds had very high potency on the latently infected cell line (Fig. 1a). The compounds EK-2, EK-6, EK-8D, EK-15A, EK-16A, EK-25D, EK-42B and EK-43B showed more than 50% of the C11 cells to be GFP positive and were further evaluated with optimized concentrations. All these compounds demonstrated a dose-dependent manner in reactivating HIV-1 (Fig. 1b). Among them, EK-16A was found to have the highest potency and was hence selected as a candidate for further investigation.

### EK-16A induces expression of HIV-1 in latently infected cells

**Figure 2:**
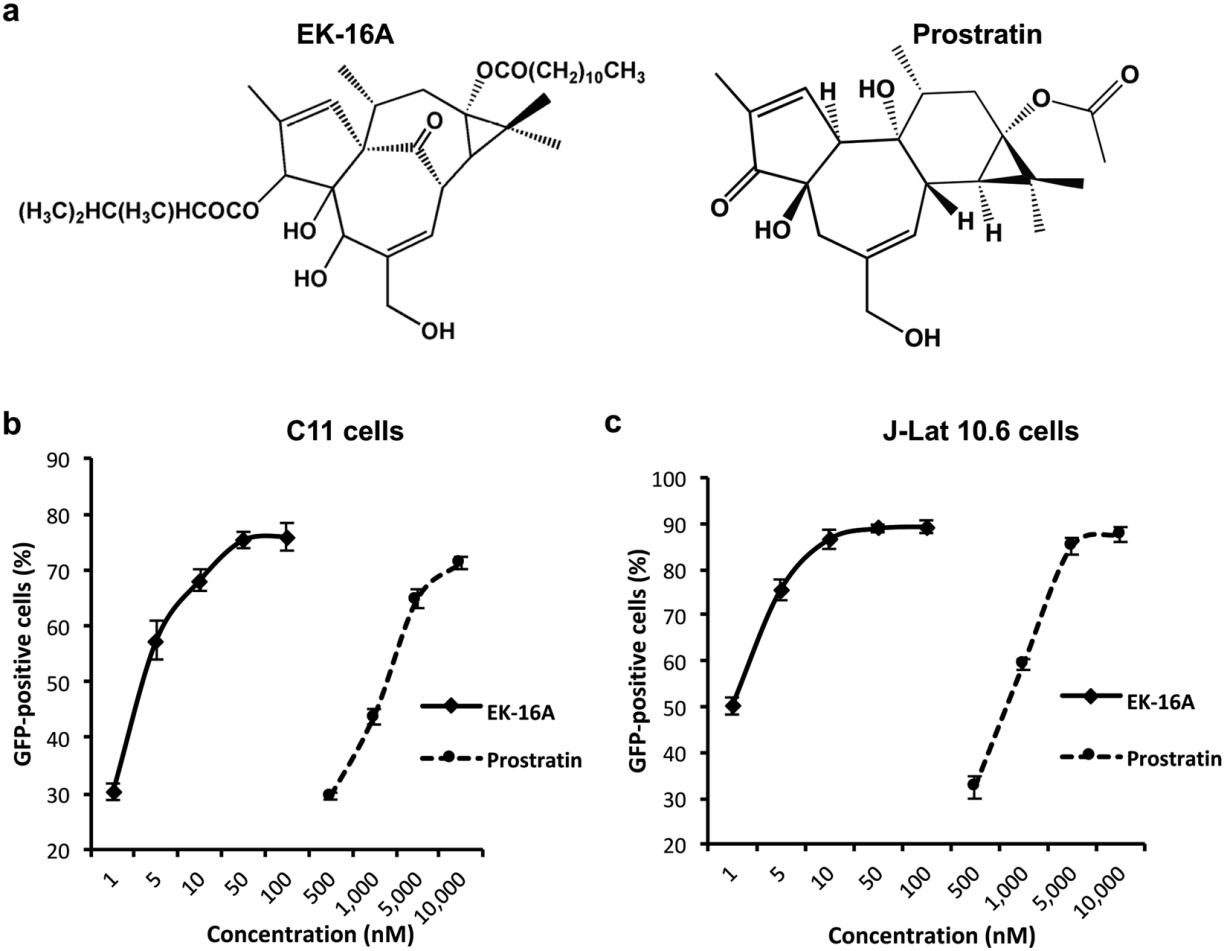
Reactivation of latent HIV-1 in latently infected cells by EK-16A and prostratin. (**a**) The structure of EK-16A and prostratin. (**b**) C11 cells and (**c**) J-Lat 10.6 cells were treated with EK-16A or prostratin at the indicated concentrations. At 48 h post-treatment, the percentage of GFP-positive cells was measured by flow cytometry and dose-dependent curves were shown. Data show the means ± s.d. of three independent experiments.

EK-16A is an ingenol ester which shares the core structure with other ingenol esters reported to reactivate latent HIV-1^37–39, 58^, but it has different side chains (Fig. 2a). These ingenol derivatives are also structurally analogous to prostratin, a phorbol ester which is known to reactivate HIV-1 from latency^33, 59^. Therefore, we first compared the latency reactivation efficiency of EK-16A and prostratin.

**Figure 3:**
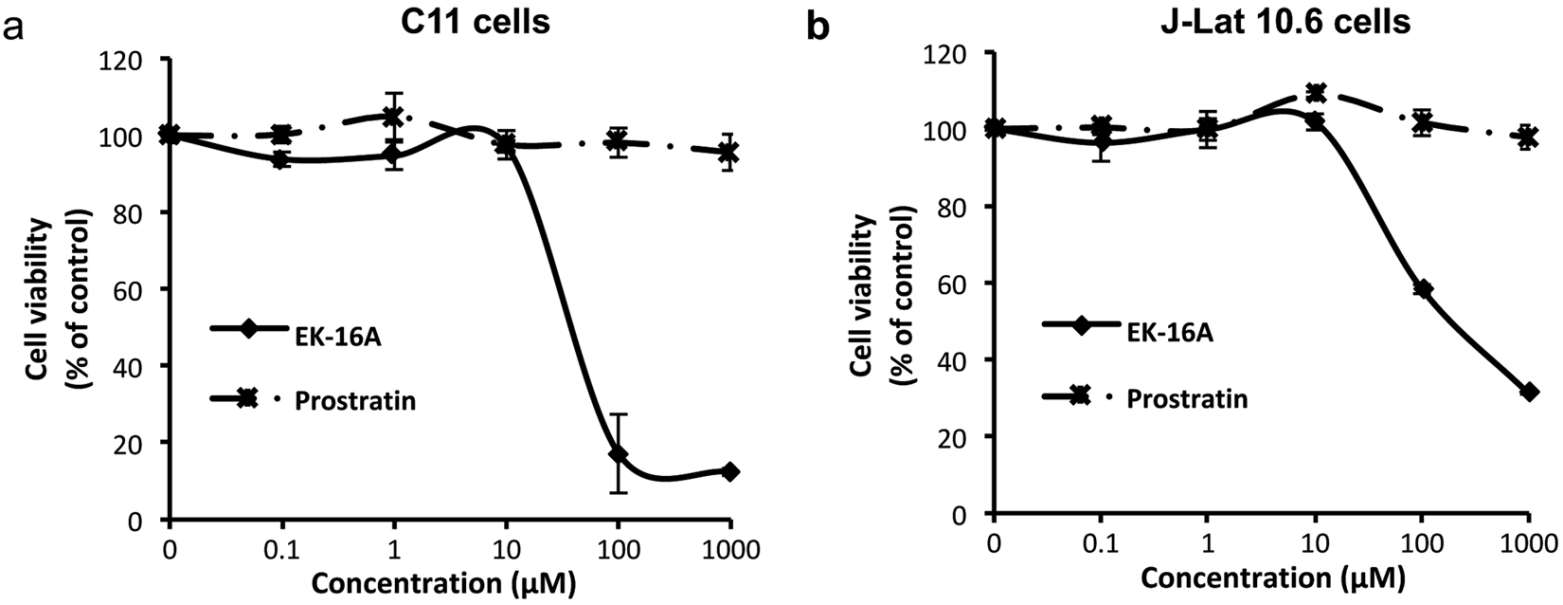
Effects of EK-16A on cell viability. C11 cells (**a**) and J-Lat 10.6 cells (**b**) were treated with EK-16A or prostratin at the indicated concentration for 72 h and then cell viability was measured by CCK-8 kit (Dojindo). The division of OD450 between treated and control groups indicate the percentage of cell viability. Data show the means ± s.d. in three independent experiments.

In order to do this, two HIV-1 latency cell culture models, C11 cells and the J-Lat 10.6 cells^60^, were used in this study. Treatment with EK-16A effectively induced GFP expression in about 80% of C11 cells and 90% of the J-Lat 10.6 cells. Both EK-16A and prostratin showed dose-dependent activity in the assay, but displayed varying levels of potency in activating HIV-1 expression (Fig. 2b,c). The dose-reponse curve of HIV-1 induction on C11 cells and J-Lat 10.6 cells indicate a mean EC_50_ value of 3.53 nM on C11 and 4.06 nM on J-Lat 10.6 for EK-16A. This was about 200-fold lower than the mean EC_50_ value of 768 nM on C11 and 865 nM on J-Lat 10.6 for prostratin as shown in Table 1, implying that EK-16A is much more potent than prostratin. Cytotoxicity measurements under similar conditions demonstrated that EK-16A caused minimal cellular toxicity on cells and had minor effects on cell proliferation at its effective concentrations (1–100 nM) (Supplementary Fig. 2). Calculations for CC_50_ value according to the cell viability-dose cure at high concentrations (Fig. 3) showed EK-16A to have a CC_50_ value of 68.51 μM on C11 cells and a CC_50_ value of 94.17 μM on J-Lat 10.6 cells, resulting in approximately 20,000-fold selectivity windows on both of these cells (Table 1). Our data showed that EK-16A is an effective activator of HIV-1 from latency without exerting detectable cellular cytotoxicity.

**Table 1:** The EC_50_ and CC_50_ values of EK-16A and prostratin on latently infected cells.

	EC_50_ (nM)		CC_50_ (nM)	
	C11	J-Lat 10.6	C11	J-Lat 10.6
EK-16A	3.53	4.06	68,510	94,170
Prostratin	768	865	>1000,000	>1000,000

### EK-16A in combination with other LRAs induce synergistic reactivation of HIV-1

**Figure 4:**
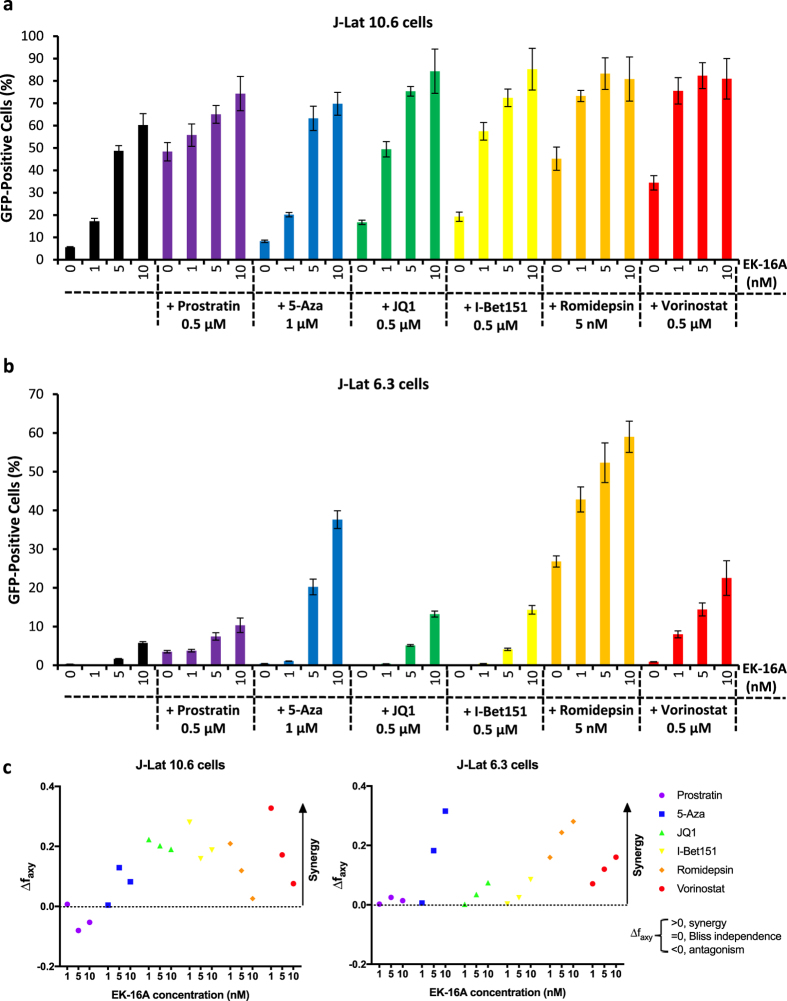
EK-16A synergizes with other LRAs in reactivating latent HIV-1. J-Lat 10.6 (**a**) and J-Lat 6.3 cells (**b**) were treated with EK-16A at the indicated concentrations alone or in combination with prostratin (0.5 μM), 5-Aza (1 μM), JQ1 (0.5 μM), I-Bet151 (0.5 μM), romidepsin (5 nM), or vorinostat (0.5 μM) for 24 h and and the percentage of GFP-positive cells was measured. (**c**) The Bliss independence model was utilized for calculation of synergy for LRA combinations (See materials and methods). Dotted horizontal line signifies pure additive effect (Δf_axy_ = 0). Synergy is defined as Δf_axy_ > 0 while Δf_axy_ < 0 indicates antagonism. Data show the means ± s.d. of three independent experiments.

Because the establishment and maintenance of HIV-1 latency involve multiple mechanisms, a single LRA may not enough to reverse latency from all the cells. Almost all the identified candidate LRAs were only minimally active in resting CD4^+^ T cells from infected individuals^48^; however, when mechanistically distinct LRAs were tested together, multiple combinations that effectively reverse latency have been identified^39, 61, 62^. To assess the synergy of EK-16A with other LRAs, one PKC agonist (prostratin), one DNA methyltransferase (DNMT) inhibitor (5-azacytidine, 5-Aza^63^), two BET inhibitors (JQ1 and I-Bet151^64^) and two HDAC inhibitors (romidepsin and vorinostat) were co-administered with EK-16A. We measured the percentage of GFP-positive cells as the marker of HIV-1 latency reactivation when J-Lat 10.6 cells were treated with EK-16A alone or in combination with the other LRAs (Fig. 4a). The same combination treatments were also performed on another latently infected cell line, J-Lat 6.3 cells^60^, which showed less responsiveness to single LRAs (Fig. 4b).

To quantify the synergy mediated by EK-16A and other LRAs, we compared the experimentally observed combined effects in both J-Lat 10.6 and 6.3 cells to the effects predicted under the Bliss independence model for combined drug effects (Fig. 4c). On both cell lines, we found that EK-16A demonstrated synergy with 5-Aza, JQ1, I-Bet151, romidepsin and vorinostat, but not with prostratin. On J-Lat 10.6 cells, lower concentrations of EK-16A showed better synergy than higher concentrations. We anticipate the potency of EK-16A on this cell line to be saturated at higher concentrations and thereby conceal any additive effects of the combination. These results are more discernable in light of the effects of EK-16A on J-Lat 6.3 cells where only the higher concentrations (5–10 nM) of EK-16A could induce some measure of HIV-1 production on this cell line (Fig. 4c) and hence a synergy was also observed at those doses when combination studies were performed. All these results demonstrate that EK-16A exerts synergic effects with DNMTis, BETis and HDACis on reactivation of HIV-1 from latently infected cell lines.

### EK-16A induces HIV-1 expression in resting CD4^+^ T cells from cART–treated patients

**Figure 5:**
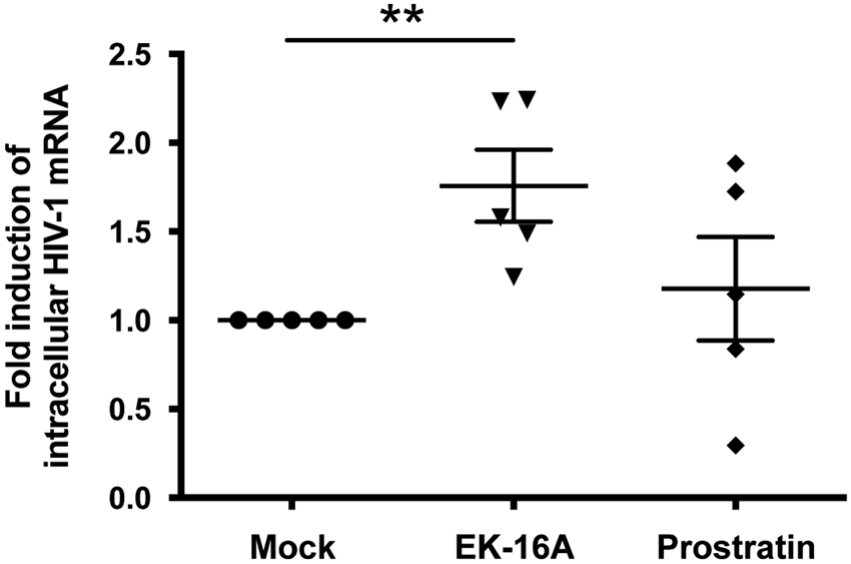
EK-16A reactivates HIV-1 from infected resting CD4^+^ T cells *ex vivo*. Resting CD4 T cells isolated from five cART-suppressed HIV-1-infected patients were treated with either EK-16A (0.05 μM) or prostratin (1 μM) for 18 h. Intracellular HIV-1 mRNA levels were detected by qRT-PCR and presented as fold induction relative to mock-treated control. **p < 0.01, two-tailed unpaired Student *t* test, n = 5.

We investigated the effect of EK-16A in *ex vivo* cultures of cells directly isolated from HIV-1–infected individuals receiving suppressive cART (Table 2). Resting CD4^+^ T cells, with a specific pattern of surface markers (including CD4^+^, CD25^−^, CD69^−^ and HLA DR^−^), represent the major reservoir for HIV-1^65^. Purified resting CD4^+^ T cells from five patients were treated with indicated doses of EK-16A or prostratin, and cellular HIV-1 reactivation examined by HIV-1 mRNA expression using qRT-PCR with primers/probe targeting HIV-1 3′ polyadenylation (poly A) region^66^. The results showed that EK-16A treatment induced full length HIV-1 transcripts in cells from all 5 donors, with a mean induction of about 2-fold whereas prostratin only increased HIV-1 expression in 3 of the 5 donors, with lower induction fold (Fig. 5). Moreover, EK-16A treatment also increased HIV-1 RNA in the supernatant of the cell cultures (Supplementary Fig. 3). These results confirm that EK-16A is able to reactivate transcription of HIV-1 from the *ex vivo* resting CD4^+^ T cells of HIV-1-infected patients.

**Table 2:** Clinical and biological characteristics of the patients in this study.

Patients	Gender	Age (years)	CD4 count at the time of the study (cells/ mm^3^)	Antiviral therapy at the time of the study	Duration of therapy (years)	Duration with undetectable plasma HIV-1 RNA level (<40 copies/ml of plasma) (months)
1	Male	51	**1372**	**AZT** + **3TC** + **NVP**	9	105
2	Male	52	**576**	**AZT** + **3TC** + **EFV**	4	25
3	Male	57	**546**	**AZT** + **3TC** + **EFV**	2	19
4	Male	45	**370**	**AZT** + **3TC** + **NVP**	9	87
5	Male	53	**576**	**AZT** + **3TC** + **EFV**	4	34
6	Male	35	**680**	**AZT** + **3TC** + **EFV**	6	8
7	Male	28	**476**	**AZT** + **3TC** + **NVP**	5	50

Note: AZT, Zidovudine; 3TC, lamivudine; NVP, nevirapine; EFV, efavirnez.

### EK-16A displays minimal toxicity in primary CD4^+^ T cells

**Figure 6:**
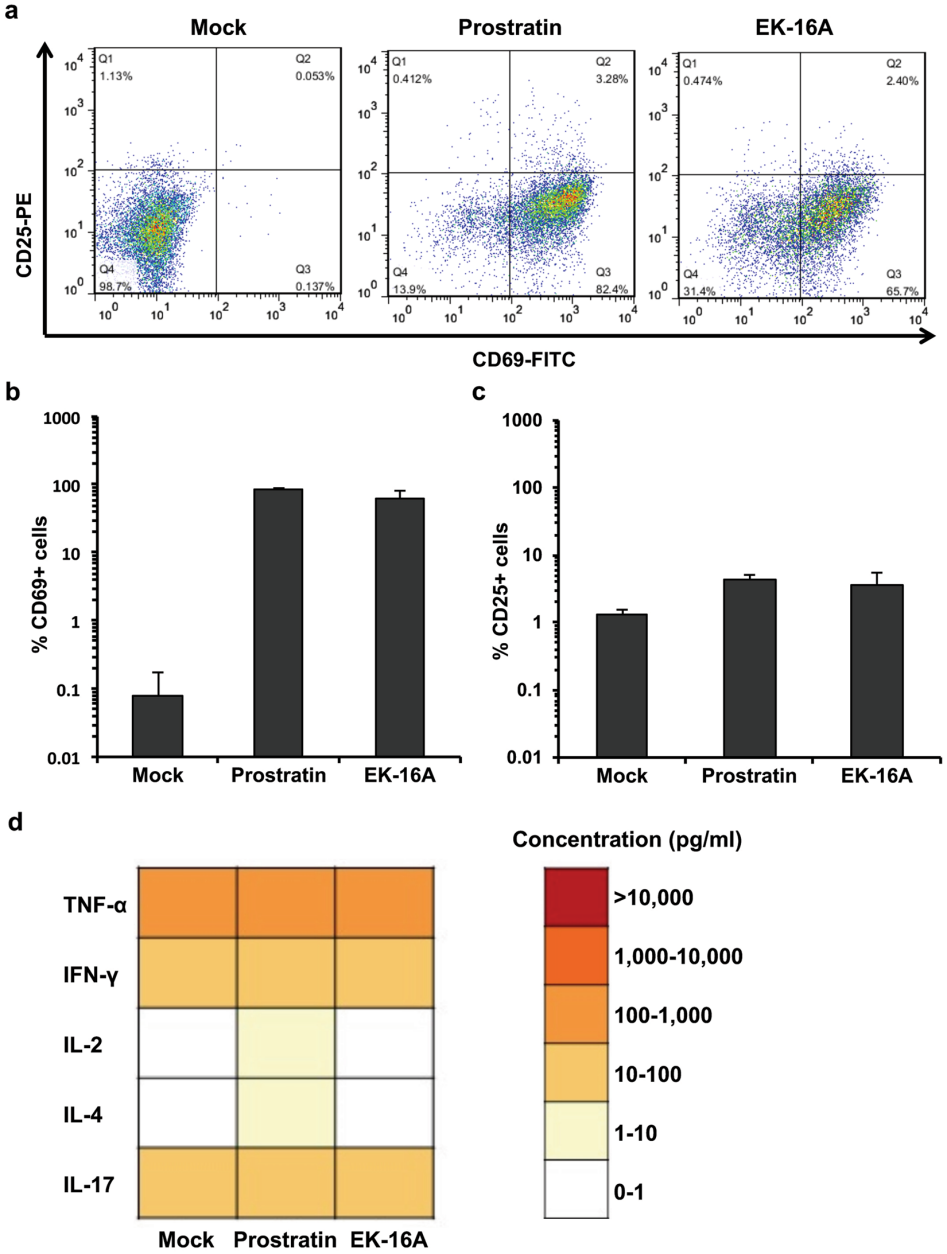
The effects of EK-16A on T cell activation. (**a**) The effect of EK-16A and prostratin on the expression of CD25 and CD69. Human CD4^+^ T cells were treated with either EK-16A (0.05 μM) or prostratin (1 μM) for 24 h and the expression of CD25 and CD69 was detected by flow cytometry using antibodies against CD25 and CD69. The percentage of CD69-positive cells (**b**) and CD25-positive cells (**c**) was calculated. Data show the means ± s.d. of three independent experiments. (**d**) Human CD4^+^ T cells were treated with EK-16A (0.05 μM) or prostratin (1 μM) for 24 h and cell culture supernatant was assayed for cytokine concentrations (pg/ml) by ELISA. Data are representative of three independent experiments.

To be clinically applicable, effective LRAs should have high potency, and should also be minimally cytotoxic without inducing global T cell activation^39, 58^. Therefore, we asked whether robust induction of latent HIV-1 by EK-16A was coupled to T cell activation or toxicity. Purified primary CD4^+^ T cells were treated with EK-16A or prostratin for 24 hours and the expression of global T cell activation maker CD25 and CD69 was measured by flow cytometry. Similar to prostratin, EK-16A treatment was associated with increased surface expression of CD69, an inducible glycoprotein that is expressed early during activation of T lymphocytes (Fig. 6a,b). For another activation marker CD25, we could see both prostratin and EK-16A have some promotion on its expression, but to a much less extent (Fig. 6a,c). We also examined a more direct effect of functional T cell activation concerning their potential toxicity by measuring the production and release of pro-inflammatory cytokines. With respect to the tested cytokines, prostratin appeared to minimally induce IL-2 and IL-4 expression, but no apparent increase in levels of other cytokines such as TNF-α, IFN-γ or IL-17. Contrary to this, EK-16A showed no significant increase in the expression of any of the tested cytokines (Fig. 6d). Similar results were reported by Laird *et al*., that prostratin treatment caused little or no cytokine production from both resting CD4^+^ T cells and PBMCs, while PMA/I treatment induced the production and release of high levels of multiple cytokines^61^. In summary, despite of increasing CD69 expression, EK-16A showed no significant impact on pro-inflammatory cytokines, suggesting that it may be a potential LRA candidate for further evaluation.

### Reactivation of HIV-1 by EK-16A depends mainly on PKCγ

**Figure 7:**
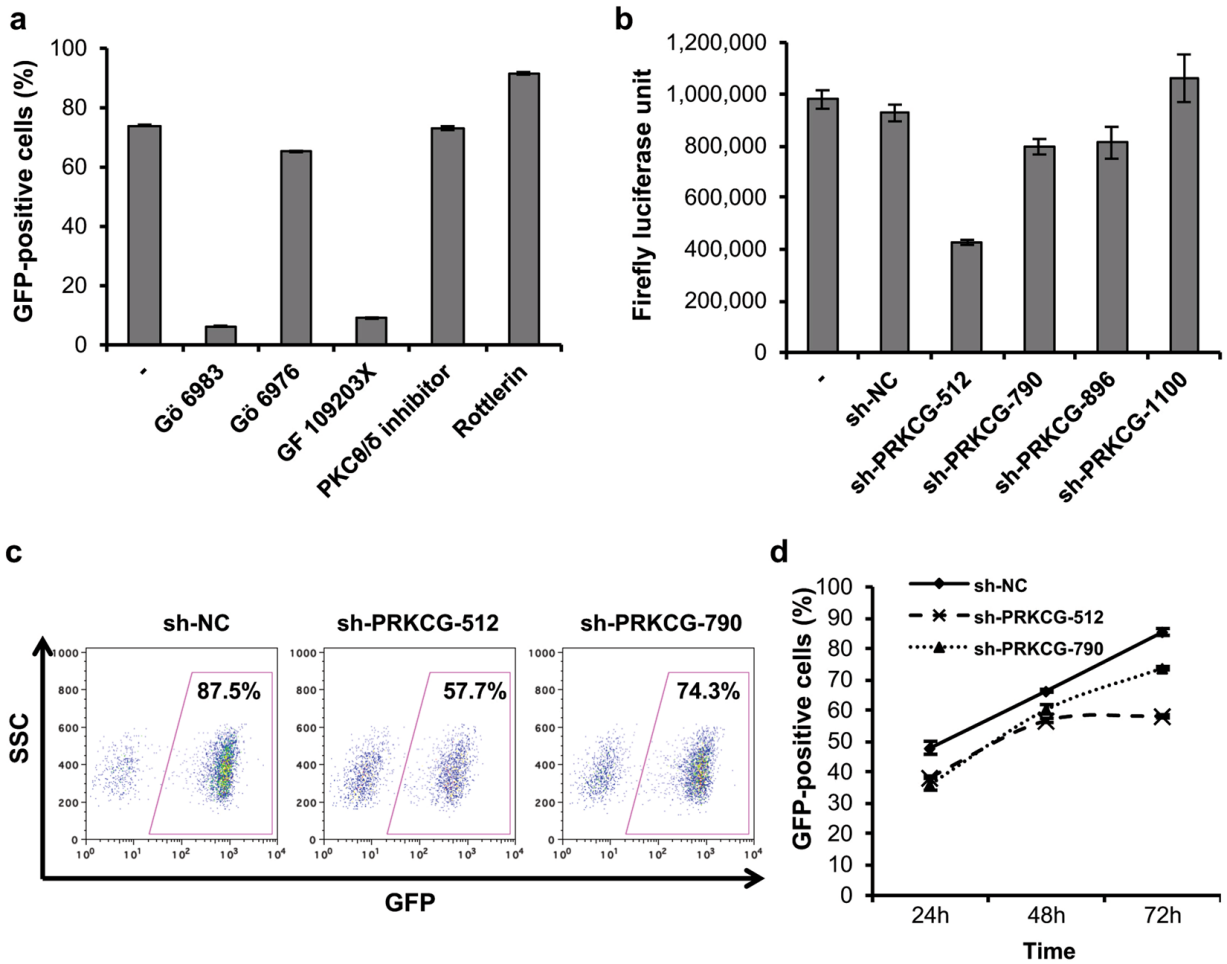
EK-16A activates HIV-1 mainly through PKCγ. (**a**) C11 cells were treated with 0.05 μM EK-16A alone (−), or pretreated with Gö 6983 (1 μM), Gö 6976 (1 μM), GF 109203X (1 μM), PKCθ/δ inhibitor (10 μM) or Rottlerin (1 μM) for 3 h, then treated with EK-16A for 48 h before percentage of GFP-positive cells was assessed by flow cytometry. (**b**) TZM-bl cells were untransfected (−) or transfected with negative control shRNA plasmid (sh-NC) or shRNA plasmids targeting different sequences of PKCγ for 24 h, then treated with 0.05 μM EK-16A for 48 h before firefly luciferase activity was measured. (**c**) C11 cells were nucleofected with indicated shRNA plasmids for 24 h, then treated with 0.05 μM EK-16A for 72 h before percentage of GFP-positive cells in the shRNA-expressing cells (RFP-positive) was measured by flow cytometry; (**d**) shows the corresponding data at the different indicated time points. All data represent the mean ± s.d. of three independent experiments.

Previous reports showed that ingenol derivatives activate HIV-1 through the Protein Kinase C (PKC) pathway, and we have determined that EK-16A belongs to the ingenol family. To further our insight into the mechanism of this potent compound, we evaluated the activation of PKC by EK-16A and examined the isoform of the enzyme involved. C11 cells were pretreated with known PKC inhibitors and then treated with EK-16A. Pretreatment with Gö 6983^67^ (a pan-PKC inhibitor against for PKCα, PKCβ, PKCγ, PKCδ, PKCζ and PKCμ) and GF 109203X (a potent and selective inhibitor of PKCα, PKCβ and PKCγ) significantly suppressed the activation of HIV-1 by EK-16A. On the other hand, a PKCα and PKCβ specific inhibitor Gö 6976^68^, a PKCθ/δ inhibitor^69^ and Rottlerin^70^ (reported as PKCδ inhibitor) showed no such potent suppression (Fig. 7a). Thus, we can infer that PKCγ may play an important role in the EK-16A reactivation of latent HIV-1.

To confirm the role of PKCγ in the reactivation of latent HIV-1 by EK-16A, we designed four short hairpin RNAs (shRNAs) targeting different sites of PKCγ gene (*PRKCG*) to knockdown its expression (Table 3). TZM-bl cells transfected with these shRNA expression plasmids were treated with EK-16A, and HIV-1 LTR driven luciferase expression detected. Compared to the untreated control, EK-16A promoted the HIV-1 LTR driven luciferase expression to approximately 10-fold. Downregulation of PKCγ by one of the shRNAs, sh-PRKCG-512 significantly suppressed luciferase expression by about 50%. Another two shRNAs, sh-PRKCG-790 and sh-PRKCG-896, also weakened the induction of LTR by EK-16A, but to a lesser extent (~20%) (Fig. 7b).

**Table 3:** Sequences of shRNAs targeted against different sites on the PKCγ gene.

shRNA	Sequence (5′-3′)
sh-PRKCG-512	GCCACGAATTTGTGACCTTCG
sh-PRKCG-790	GCAGATGAGATCCACGTAACT
sh-PRKCG-896	GGAACCTGACGAAACAGAAGA
sh-PRKCG-1100	GCTGGTACAAGTTACTGAACC

Using two of these shRNAs, sh-PRKCG-512 and sh-PRKCG-790, we further confirmed the role of PKCγ in HIV-1 reactivation in C11 cells. C11 cells nucleofected with selected shRNAs were treated with EK-16A and GFP expression was monitored as a read-out of latent HIV-1 reactivation. Expression of shRNAs was monitored by presence of red fluorescence protein (RFP) (Supplementary Fig. 4, plasmid map) and shRNA-expressing (RFP-positive) cells were gated using flow cytometry. Our results demonstrate that knockdown of PKCγ by both sh-PRKCG-512 and sh-PRKCG-790 impeded the reactivation of latent HIV-1 by EK-16A. While absence of a shRNA expression induced reactivation in about 87% of the cells after EK-16A addition, only 57% and 74% of the sh-PRKCG-512- and sh-PRKCG-790-transfected cells, respectively, could be reactivate by the same EK-16A treatment (Fig. 7c,d). Taking these results into account, we can claim an important role for the PKCγ enzyme involved pathway in reactivating HIV-1 in cells receiving EK-16A.

### EK-16A upregulates both NF-κB and P-TEFb

**Figure 8:**
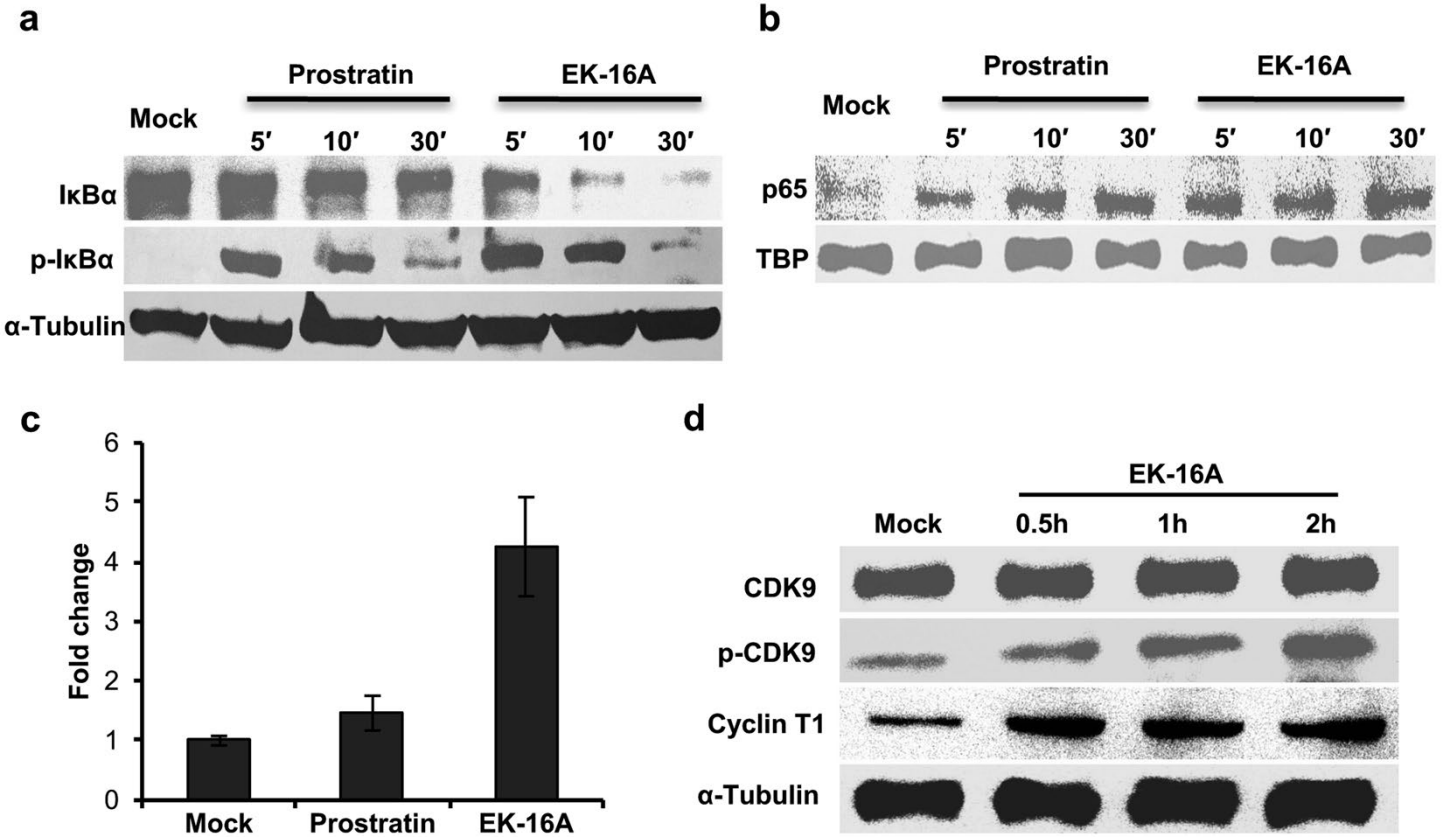
EK-16A activates HIV-1 by up-regulation of NF-κB and P-TEFb. C11 cells were stimulated with prostratin (1 μM) or ΕΚ-16Α (0.05 μM) for 5, 10 or 30 min, and (**a**) total cell lysates were probed for the expression of total IκBα or phosphorylated IκBα (p-IκBα) in immunoblots. α-Tubulin expression was used as a control for protein loading. (**b**) Nuclear extracts were analyzed using antibodies against p65. TATA-binding protein (TBP) expression was used as a control for protein loading. (**c**) C11 cells were mock-treated or stimulated with prostratin (1 μM) or ΕΚ-16Α (0.05 μM) for 30 min. ChIP assays were performed using anti-p65 antibody and PCR primers for the LTR promoter. The percentage of input for each immunoprecipitation was calculated and the fold occupancy of p65 relative to mock-treatment is shown. Each ChIP experiment was repeated three times to confirm reproducibility of results. (**d**) C11 cells were stimulated with 0.05 μM EK-16A for 0.5, 1 or 2 h, and total cell lysates were probed for the expression of total CDK9, phosphorylated CDK9 (p-CDK9) or Cyclin T1 in immunoblots. α-Tubulin expression was used as a control for protein loading.

Having demonstrated the involvement of PKC pathway in reactivation of HIV-1 by EK-16A, we wanted to further our knowledge on host proteins, especially transcription factors that would be differentially expressed in response to treatment of the cell by this novel molecule. Many reports about reactivating latent HIV-1 involving PKC agonists have implicated nuclear factor kappa B (NF-κB) as a major contributor to the transcription^58^. In unstimulated cells, NF-κB was retained in an inactive form by binding to the inhibitor of NF-κB proteins (IκBs) in the cytosol. The classical signaling cascade leading to NF-κB activation depends on phosphorylation-induced proteosomal degradation of IκBs^71^. Therefore, we then asked whether EK-16A treatment can promote the degradation of IκBs and the nuclear translocation of NF-κB. To answer this question, we treated C11 cells with EK-16A and prostratin for different times, and Western blot was performed to detect the IκBα protein levels. Compared to the mock-treated cells, both prostratin- and EK-16A-treated cells showed obvious degradation of IκBα. However, the degradation of IκBα induced by prostratin was less complete than that induced by EK-16A (Fig. 8a, upper lane). Furthermore, we confirmed that the degradation of IκBα is related to its phosphorylation. Soon (5–10 min) after the stimulation, high level of phosphorylated IκBα proteins could be detected in the cell lysates (Fig. 8a, middle lane). We then monitored the NF-κB p65 protein content in the nucleus after drug treatment. Similar to prostratin treatment, treating cells with EK-16A for 5 to 30 minutes significantly increased the amount of the NF-κB p65 protein in the nucleus (Fig. 8b). We also observed the EK-16A-induced nuclear translocation of p65 by immunofluorescence microscopy (Supplementary Fig. 5).

To investigate whether NF-κB p65 was directly recruited to the HIV-1 LTR after translocating to the nucleus, we further performed ChIP assays. Chromatin fragments from C11 cells mock-treated or treated with prostratin or EK-16A were immunoprecipitated with anti-p65 antibodies or normal mouse IgG. DNA was isolated from the immunoprecipitates and was analyzed by PCR using primers specific for HIV-1 LTR. We observed that the amounts of p65 bound to the LTR region were increased following both prostratin or EK-16A treatment. When the fold occupancy of p65 relative to mock-treatment was calculated, the doses of EK-16A tested induced greater than 4-fold levels of p65 recruited to LTR, much higher than the induction of prostratin doses tested (Fig. 8c). These results suggest that EK-16A can promote NF-κB translocate to the nucleus and induce the transcription initiation from HIV-1 LTR.

PKC agonists have also been reported to upregulate P-TEFb expression and phosphorylation^34, 72, 73^. Therefore, we first investigated the effect of EK-16A on CDK9, one component of P-TEFb. C11 cells were treated with EK-16A for different times and the content of CDK9 and phosphorylated CDK9 proteins was measured. CDK9 expression was not induced by EK-16A treatment; however, a significant induction of CDK9 T-loop (Thr-186) phosphorylation was observed as early as 30 min after stimulation (Fig. 8d). For the other component of P-TEFb, Cyclin T1, we could detect apparent expression induction by EK-16A treatment at both RNA and protein levels (Fig. 8d and Supplementary Fig. 6). Together, these results indicated that EK-16A promoted the HIV-1 transcription elongation through an upregulation of P-TEFb by increasing CDK9 T-loop phosphorylation and Cyclin T1 levels.

**Figure 9:**
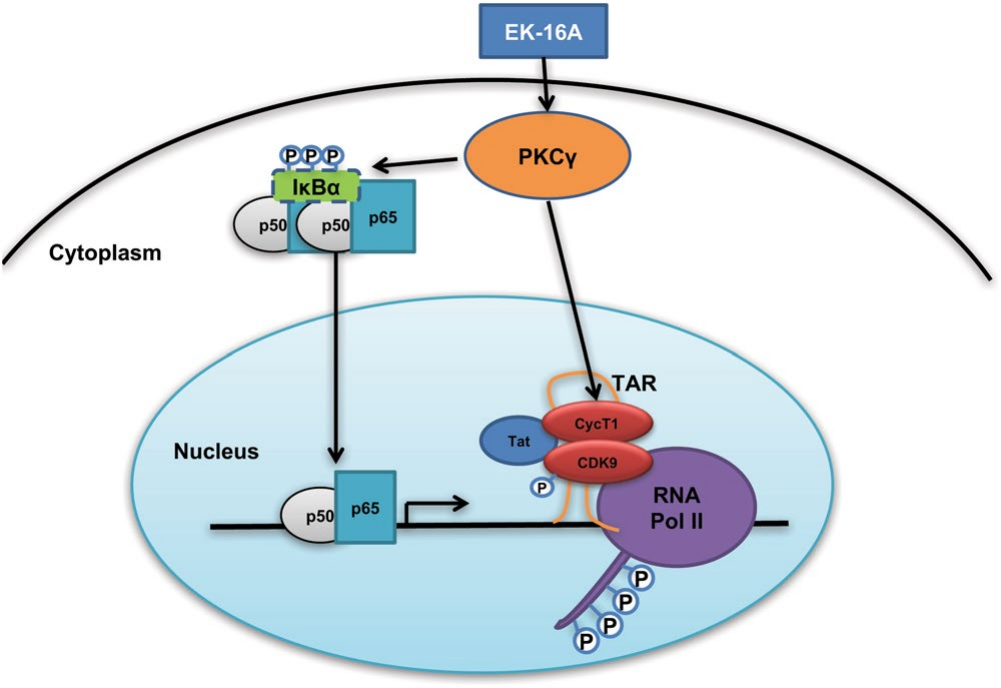
Diagram depicting EK-16A-mediated HIV-1 reactivation by both NF-κB and P-TEFb signaling pathways.

Based on our cumulative data, we summarize the signaling pathways in EK-16A-mediated regulation of HIV-1 gene expression (Fig. 9). Following the activation of PKC by EK-16A, IκBs proteins are phosphorylated and degradated, allowing NF-κB translocation into the nucleus and binding to HIV-1 LTR, which promotes HIV-1 transcription initiation. On the other hand, EK-16A also promotes transcription elongation by induction of P-TEFb. Because it can affect both transcription initiation and transcription elongation, EK-16A is likely capable of reversing HIV-1 latency more efficiently and at lower doses than prostratin.

## Discussion

*Euphorbia kansui* has been widely used in traditional Chinese medicine with a wide range of pharmacological activities including, but not limited to, tumor inhibition^74^, anti-viral effects^75, 76^ and regulation of immune system^77, 78^. Using HIV-1 latently infected C11 cells, we screened out more than 20 active compounds from *Euphorbia kansui* that can potently antagonize HIV-1 latency. Our results of the screening led us to down-selecting the molecule with highest potency in reversing HIV-1 latency for further investigation. Regarding the other compounds, their characterization will be addressed during follow-up studies.

The main active ingredient of *Euphorbia kansui* are diterpene esters, mainly ingenols that have significant anticancer and antiviral activities^79, 80^. Among them, ingenol mebutate is a topical drug approved by FDA for the treatment of actinic keratosis^81, 82^. Another ingenol derivative, ingenol 3-angelate (I3A) has been found to regulate HIV-1 expression for many years^83–85^. More recently, ingenol B from *Euphorbia tirucalli* was reported to reactivate latent HIV-1^37, 38, 86^. The compound selected in this study, EK-16A, also belongs to the class of ingenol derivatives. We found that EK-16A can significantly reactivate HIV-1 from latency at nanomolar concentrations in latently infected Jurkat cell lines, with much higher potency than prostratin. Notably, the effect of EK-16A on reactivation of latent HIV-1 could be further enhanced by combination with other classes of LRAs. In addition to the *in vitro* effects, we found that EK-16A could promote HIV-1 expression in the *ex vivo* resting CD4^+^ T cells from patients on supressive cART. These results encouraged us to investigate the mechanism of action that underlies this compound’s role as a potential latency-reversing candidate.

EK-16A belongs to ingenol esters, which are structurally analogous to prostratin (phorbol ester), and both of them are PKC activators^87^. The PKC family of protein serine/threonine kinases are widely distributed in cells and play crucial roles in several signal transduction cascades^88, 89^. There are at least ten PKC isoforms which can be subdivided into three subfamilies based on their domain structure and cofactor requirement. The classical or conventional PKC isoforms (α, βI, βII and γ) can be regulated by phospholipids and diacylglycerol (DAG) as well as Ca^2+^; novel PKC isoforms (δ, ε, θ, η and μ) can be regulated by phospholipids and DAG but not by Ca^2+^; while atypical isoforms (ζ and ι/λ) do not respond to either DAG or Ca^2+^, but can be regulated by phospholipids^88–91^. Thus, these PKC isoforms serve different biological roles in various signal pathways.

To identify the pertinent isoform involved in the reactivation of HIV-1 by EK-16A, we tested several PKC inhibitors with selective inhibition of different isoforms and found that only those inhibitors that suppressed PKCγ activity could significantly impede the promotion of HIV-1 by EK-16A. Moreover, knockdown of PKCγ using the specifically designed shRNA also hindered the EK-16A-mediated HIV-1 reactivation, which further verified the critical role of this PKC isoform. Another ingenol derivative, ingenol-3-hexanoate was reported by Jiang *et al*. to reactivate latent HIV-1 expression through PKCδ signaling^38^. However, Pandelo Jose *et al*. reported that the same molecule 3-caproyl-ingenol, reactivated latent HIV via activation of multiple PKC isoforms including PKCα, PKCγ and PKCδ^37^. Although the core structure of EK-16A resembles that of other ingenol esters, its different side-chains might have important roles for its activity in PKCγ activation and HIV-1 expression regulation.

The NF-κB signaling pathway is the most common downstream pathway activated by PKC agonists^58^. In resting cells, NF-κB is sequestered in the cytoplasm as a p65/p50 heterodimer in an inactive form through interactions with IκBα. Activated PKC can induce IKK-mediated phosphorylation of IκBα followed by its ubiquitylation and degradation. The released p65/p50 translocate to the nucleus and replace p50/p50 bound to LTR, resulting in transcriptional activation^11, 58, 92^. In this study, we detected the degradation of IκBα in the cytoplasm after EK-16A treatment and we also observed the nuclear translocation of p65/p50 as well as its binding to HIV-1 LTR. Our results confirmed that EK-16A promotes HIV-1 transcription initiation through PKC directed NF-κB signal pathway. On the other hand, we also found the promotion effects of EK-16A on the phosphorylation and expression of P-TEFb complex. After the earlier initiation phase, viral protein Tat is made in the HIV-1 infected cells, which can recruit P-TEFb to transactivation-responsive (TAR) region of LTR^93^. As part of the P-TEFb complex, CDK9 can phosphorylate the CTD of RNAP II and enhance its processivity^94^. CDK9 also phosphorylates subunits of two negative factors that associate with the RNAP II complex – DSIF (DRB Sensitivity Inducing Factor) and NELF (Negation Elongation Factor), and thereby counters their negative function^73, 95^. For all these catalytic activities of CDK9, its T-loop phosphorylation is essential^73^ and EK-16A does demonstrate the induction of the phosphorylation in our studies. Various other signaling factors including MAPK and AP-1 that may likely be involved in the downstream pathways following the PKC activation^96^ leading to HIV-1 activation, need to be further verified. However, in this study, we already confirmed that EK-16A can simultaneously promote the HIV-1 transcriptional initiation and elongation through NF-κB and P-TEFb, providing a viable rationale for the high efficacy of EK-16A in reactivating latent HIV-1.

PKC agonists, though promising in inducing latency reversal, lead to global T cell activation and some even result in the release of pro-inflammatory cytokines^59, 61, 97^. However, EK-16A shows no significant promotion of pro-inflammatory cytokines release, suggesting it to be a good LRA candidate. Combination of EK-16A with other LRAs permitted the use of a lower effective dose, which is likely to further reduce the toxic side effects. Moreover, researchers from different groups recently reported that addition of ruxolitinib^97^ (a kinase inhibitor) or rapamycin^98^ (an inhibitor of the mammalian target of rapamycin) could decrease T cell activation-induced pro-inflammatory cytokine release without significantly reducing latency reversal, thereby providing a possible incentive to overcome the major barrier to using ingenol compounds in clinical trials.

In summary, we have isolated a substantially potent ingenol derivative from *Euphorbia kansui*, and proven that it is a PKC agonist that induced both NF-κB and P-TEFb, with great potency in reversing HIV-1 latency. This compound could become a useful addition to the armamentarium for the permanent cure of HIV-1 infection.

## Materials and Methods

### Cell culture

Jurkat T cells and the Jurkat-derived HIV-1 latently infected C11 cells^56, 57^, J-Lat 10.6 and J-Lat 6.3 cells^60^ were grown in RPMI 1640 medium supplemented with 10% (v/v) fetal bovine serum (FBS) (Gibco, Grand Island, NY, USA), 100 U/ml penicillin and 100 μg/ml streptomycin (Life Technologies, Gaithersburg, MD, USA) at 37 °C under 5% CO_2_. TZM-bl cells were grown in Dulbecco’s modified Eagle’s medium (DMEM) (Gibco) with 10% FBS, 100 U/ml penicillin and 100 μg/ml streptomycin at 37 °C under 5% CO_2_.

### Extraction and isolation of compounds from *Euphorbia kansui*

Dried root of *Euphorbia kansui* was refluxed with 95% ethanol for 2 h (2 times). After filtration, the combined solution was evaporated under reduced pressure and the residue was extracted by methylene chloride or petroleum ether and then evaporated to obtain an oily extraction. The extraction was chromatographed by silica gel column using stepwise gradient elution with Ethyl acetate and hexane (1:10 to 10:1) to obtain purified compounds.

### Flow cytometry

The percentage of GFP-positive cells was measured by flow cytometry to determine the level of HIV-1 expression. After incubating with the compounds at the indicated concentrations for the specified times, cells were washed and resuspended in PBS. The GFP expression was measured by FACScan (Becton Dickinson FACScan Flow Cytometer), and FACS data were analyzed using Cell Quest software (Macintosh, Sunnyvale, CA, USA). Live cells were gated and two parameter analyses were used to differentiate GFP-associated fluorescence from background fluorescence. A total of 10,000 gated events were collected and data represent the percentage of GFP-expressing cells in total gated events. T-cell activation in PBMCs was monitored after staining for activation markers with FITC conjugated anti-CD25 and PE conjugated anti-CD69 antibodies. Briefly, 1 × 10^6^ cells were washed with PBS and stained with 10 μl of fluorescently labeled antibodies in 100 μl PBS containing 1% FBS for 30 min on ice. Subsequently, cells were washed three times with PBS and finally resuspended in 1 ml PBS containing 1% FBS and 10,000 cells were acquired using FACScan with Cell Quest software. All experiments were performed independently at least three times in triplicate per experimental point.

### Quantitative analysis of latency-reversing agent combinations

We used the Bliss independence model as described previously^41, 61^ to evaluate the latency-reversing activity of drug combinations. This model is defined by the equation *fa*_*xy*,*P*_ = *fa*_*x*_ + *fa*_*y*_ − (*fa*_*x*_)(*fa*_*y*_), where *fa*_*xy*,*P*_ is the predicted fraction affected by a combination of drug *x* and drug *y*, given the experimentally observed fraction affected by treatment with drug *x* (*fa*_*x*_) or drug *y* (*fa*_*y*_) individually. The experimentally observed fraction affected by a combination of drug *x* and drug *y* (*fa*_*xy*,*O*_) can be compared with the predicted fraction affected, which is computed using the Bliss model (*fa*_*xy*,*P*_) as follows: ∆*fa*_*xy*_ = *fa*_*xy*,*O*_ − *fa*_*xy*,*P*_.

If Δ*fa*_*xy*_ > 0 with statistical significance, then the combined effect of the two drugs exceeds that predicted by the Bliss model and the drug combination displays synergy. If Δ*fa*_*xy*_ = 0, then the drug combination follows the Bliss model for independent action. If Δ*fa*_*xy*_ < 0 with statistical significance, then the combined effect of the two drugs is less than that predicted by the Bliss model and the drug combination displays antagonism. In our analysis, the fraction affected was calculated as follows for the percentage of GFP-positive cells: *fa*_*x*_ = % GFP-positive cells after treatment with drug *x*–% GFP-positive cells treated with the DMSO control.

### Isolation and culture of primary CD4^+^ T cells

Seven HIV-1–infected individuals receiving suppressive cART recruited by the Shanghai Public Health Clinical Center were included in this study. All individuals had received cART for at least 3 months, had a CD4 count >350 cells/mm^3^, and maintained blood plasma HIV-1 RNA levels lower than 40 copies/mL at the time of the study. Clinical and biological characteristics of the patients are listed in Table 2. This study was conducted with the official written approval (written form) of the Ethics Committee of Fudan University and all experiments were performed in accordance with relevant guidelines and regulations of Ethics Committee of Fudan University. Written informed consent was obtained from all subjects. Whole peripheral blood from healthy donors was purchased from the Blood Center of Shanghai. Peripheral blood mononuclear cells (PBMCs) were isolated by density gradient centrifugation applying Ficoll-Hypaque (density = 1.077 g/mL; Haoyang Biological manufacture, Tianjin, China). CD4^+^ T cells were purified using a CD4^+^ T cell Isolation Kit II (Miltenyi Biotech, Auburn, CA, USA) according to the manufacturer’s instructions. Resting CD4^+^ T cells were further isolated using anti-CD25, anti-CD69 and anti-HLA-DR antibody-coated magnetic beads (Miltenyi Biotech). For all the reactivation experiments, cells were cultured with 10 μM T20 to prevent new infection events^48^.

### RNA isolation and qRT-PCR

Total RNA was isolated using an RNeasyPlus Mini Kit (QIAGEN) following the manufacturer’s protocol. cDNA was synthesized from DNase-treated RNA using a QuantiTect Reverse Transcription Kit (QIAGEN) according to the manufacturer’s instructions. qRT-PCR amplification of duplicate cDNAs was performed using the Roche Lightcycler 480 Real-Time PCR System as described previously^48, 57, 66^. The primers and probes used for detecting the intracellular RNA were: HIV-1 mRNA forward: 5′-CAGATGCTGCATATAAGCAGCTG-3′; HIV-1 mRNA reverse: 5′-TTTTTTTTTTTTTTTTTTTTTTTTGAAGCAC-3′; HIV-1 mRNA probe: 5′-FAM-CCTGTACTGGGTCTCTCTGG-MGB-3′. The primers and probes used for detecting the supernatant RNA were specific to HIV-1 *gag* gene (gag-forward: 5′-ATCAATGAGGAAGCTGCAGAA-3′, gag-reverse: 5′-GATAGGTGGATTATGTGTCAT-3′, gag-probe: 5′-FAM-ATTGCACCAGGCCAGATGAGAGAA-TAMRA-3′). The cycling parameters of quantitative PCR were as follows: (i) 2 min at 50 °C and then 10 min at 95 °C; (ii) 40 to 45 cycles at 95 °C for 15 s, 60 °C for 60 s. Each sample was tested in triplicate, and results were normalized with the human *TATA-binding protein (TBP)* gene.

### *In vitro* cytotoxicity assay

The Cell Counting Kit-8 (CCK-8) (Dojindo, Kumamoto, Japan) was used to measure the *in vitro* cytotoxicity of the compounds. Briefly, approximately 4 × 10^4^ cells per well were treated with EK-16A or prostratin for 72 h, and then 10 μl of CCK-8 solution was added to each well of the 96-well culture plates. After 4 h of incubation at 37 °C, the absorbance at 450 nm was measured using a microplate reader. The 50% cytotoxic concentration (CC_50_) was calculated by nonlinear regression analysis using GraphPad Prism 5 software (GraphPad, SanDiego, CA).

### ELISA detection of cytokine release

Human PBMCs were treated with EK-16A (0.05 μM) or prostratin (1 μM) for 24 h and the TNF-α, IFN-γ, IL-2, IL-4 and IL-17 concentrations (pg/ml) were monitored in culture supernatant by using the specific cytokine ELISA kits (Shanghai Shuangying Biological Technology Co., Ltd., Shanghai, China) according to the manufacturer’s instructions.

### Transfection with shRNA plasmids

Knockdown of PKCγ was performed by transfection with shRNA plasmids targeting different sequences of *PRKCG* gene (Genebank Accession: NM_002739). The shRNA expressing plasmids were designed and synthesized by GenePharma (Shanghai, Chia). For TZM-bl cells, the plasmids were transfected with lipofectamine 2000 (Life Technologies, Gaithersburg, MD). For the transfection of C11 cells, an Amaxa Cell Line Nucleofector Kit V (Lonza, Gaithersburg, MD, USA) was used.

### Western blotting

C11 cells were adjusted to 1 × 10^6^ cells/ml and stimulated with 1 μM prostratin or 0.05 μM EK-16A for various time. Whole-cell extracts or the nuclear fractions were prepared as described previously^99, 100^. Proteins were separated by SDS-PAGE, transferred to nitrocellulose membrane, and visualized with primary and secondary antibodies and ECL chemiluminescence system (Santa Cruz Biotechnology, Santa Cruz, CA). The following primary antibodies were used: anti-IκBα (catalog # 9242), anti-p-IκBα (# 9246), anti-CDK9 (# 2316), anti-p-CDK9 (# 2549), anti-Cyclin T1 (# 8744), anti-α-Tubulin (# 2125), anti-TBP (# 12578) (Cell Signaling, Danvers, MA), and anti-p65 (catalog # sc-56735; Santa Cruz).

### Chromatin immunoprecipitation

ChIP assays were performed according to the manufacturer’s protocol (Millipore, Billerica, MA, USA) and a previously described procedure^100, 101^. Briefly, C11 cells were mock-treated or treated with prostratin (1 μM) or EK-16A (0.05 μM) for 30 min, then cross-linked with 1% formaldehyde, and the lysates were sonicated to produce 500–1000 bp chromatin fragments. The supernatant, which contained the precleared chromatin, was then incubated at 0 °C overnight with 2 μg of anti-p65 (Santa Cruz) or normal mouse IgG (Millipore). After the samples were reverse cross-linked by an overnight incubation at 65 °C, the DNA was isolated and analyzed by PCR for 30 cycles (using the forward 5′-AGACTGCTGACATCGAGCTTTCT-3′ and reverse 5′-GTGGGTTCCCTAGTTAGCCAGAG-3′ primers), which produced a 192 bp PCR fragment containing the NF-κB binding sites.

### Immunofluorescence Staining

Immunocytochemistry was performed as described previously^99^. Briefly, C11 cells were treated with prostratin (1 μM) or EK-16A (0.05 μM) for 30 min. After fixed with 4% paraformaldehyde (PFA) for 20 minutes and permeated with 0.5% Triton X-100 for 20 min, the samples were stained with 1/100 dilution of anti-human p65 rabbit polyclonal immunoglobulin G (IgG) (sc-71516, Santa Cruz) for 1 h, 1/200 dilution of Alexa-555-coupled goat anti-rabbit IgG (Molecular Probes) for 40 min, and then 0.5 μg/ml DAPI for 5 min. Finally, the samples were washed and saturated with PBS to be analyzed on a Zeiss Axio Observer Z1 microscope.

### Statistical analysis

Data are representative of three independent experiments, and error bars represent standard deviation. Two treatment groups were compared by the two-tailed unpaired Student *t* test, using Microsoft Excel and Prism 5.0 (GraphPad). Statistical significance was indicated at *p < 0.05, **p < 0.01 or ***p < 0.001.

## Electronic supplementary material

Supplementary Information

## References

[1] Hammer SM (1997). A controlled trial of two nucleoside analogues plus indinavir in persons with human immunodeficiency virus infection and CD4 cell counts of 200 per cubic millimeter or less. AIDS Clinical Trials Group 320 Study Team. N. Engl. J. Med..

[2] Gulick RM (1997). Treatment with indinavir, zidovudine, and lamivudine in adults with human immunodeficiency virus infection and prior antiretroviral therapy. N. Engl. J. Med..

[3] Perelson AS (1997). Decay characteristics of HIV-1-infected compartments during combination therapy. Nature.

[4] Davey RT (1999). HIV-1 and T cell dynamics after interruption of highly active antiretroviral therapy (HAART) in patients with a history of sustained viral suppression. Proc. Natl. Acad. Sci. USA.

[5] Torres RA, Lewis W (2014). Aging and HIV/AIDS: pathogenetic role of therapeutic side effects. Lab. Investig..

[6] Hearps AC, Martin GE, Rajasuriar R, Crowe SM (2014). Inflammatory co-morbidities in HIV+ individuals: learning lessons from healthy ageing. Curr. HIV/AIDS Rep..

[7] Samaras K (2009). Prevalence and pathogenesis of diabetes mellitus in HIV-1 infection treated with combined antiretroviral therapy. J. Acquir. Immune Defic. Syndr..

[8] Peterlin BM, Trono D (2003). Hide, shield and strike back: how HIV-infected cells avoid immune eradication. Nat. Rev. Immunol..

[9] Archin NM (2014). Eradicating HIV-1 infection: seeking to clear a persistent pathogen. Nat. Rev. Microbiol..

[10] Battistini A, Sgarbanti M (2014). HIV-1 latency: an update of molecular mechanisms and therapeutic strategies. Viruses.

[11] Ruelas DS, Greene WC (2013). An integrated overview of HIV-1 latency. Cell.

[12] Delagreverie HM (2016). Ongoing Clinical Trials of Human Immunodeficiency Virus Latency-Reversing and Immunomodulatory Agents. Open Forum Infect Dis..

[13] Dahabieh MS, Battivelli E, Verdin E (2015). Understanding HIV latency: the road to an HIV cure. Annu. Rev. Med..

[14] Datta PK (2016). HIV-1 Latency and Eradication: Past, Present and Future. Curr. HIV Res..

[15] Shang HT (2015). Progress and challenges in the use of latent HIV-1 reactivating agents. Acta Pharmacol. Sin..

[16] Margolis, D. M., Garcia, J. V., Hazuda, D. J. & Haynes, B. F. Latency reversal and viral clearance to cure HIV-1. *Science* **353**, aaf6517 (2016). 10.1126/science.aaf6517 PMC5021637 27463679

[17] Kulkosky J (2002). Intensification and stimulation therapy for human immunodeficiency virus type 1 reservoirs in infected persons receiving virally suppressive highly active antiretroviral therapy. J. Infect. Dis..

[18] Prins JM (1999). Immuno-activation with anti-CD3 and recombinant human IL-2 in HIV-1-infected patients on potent antiretroviral therapy. AIDS.

[19] Manson McManamy ME, Hakre S, Verdin EM, Margolis DM (2014). Therapy for latent HIV-1 infection: the role of histone deacetylase inhibitors. Antivir. Chem. Chemother..

[20] Lehrman G (2005). Depletion of latent HIV-1 infection *in vivo*: a proof-of-concept study. Lancet.

[21] Siliciano JD (2007). Stability of the latent reservoir for HIV-1 in patients receiving valproic acid. J. Infect. Dis..

[22] Archin NM (2008). Valproic acid without intensified antiviral therapy has limited impact on persistent HIV infection of resting CD4+ T cells. AIDS.

[23] Sagot-Lerolle N (2008). Prolonged valproic acid treatment does not reduce the size of latent HIV reservoir. AIDS.

[24] Archin NM (2010). Antiretroviral intensification and valproic acid lack sustained effect on residual HIV-1 viremia or resting CD4+ cell infection. PLoS One.

[25] Routy JP (2012). Valproic acid in association with highly active antiretroviral therapy for reducing systemic HIV-1 reservoirs: results from a multicentre randomized clinical study. HIV med..

[26] Archin NM (2014). HIV-1 expression within resting CD4+ T cells after multiple doses of vorinostat. J. Infect. Dis..

[27] Archin NM (2012). Administration of vorinostat disrupts HIV-1 latency in patients on antiretroviral therapy. Nature.

[28] Elliott JH (2014). Activation of HIV transcription with short-course vorinostat in HIV-infected patients on suppressive antiretroviral therapy. PLoS Pathog..

[29] Rasmussen TA (2014). Panobinostat, a histone deacetylase inhibitor, for latent-virus reactivation in HIV-infected patients on suppressive antiretroviral therapy: a phase 1/2, single group, clinical trial. Lancet HIV.

[30] Wei DG (2014). Histone deacetylase inhibitor romidepsin induces HIV expression in CD4 T cells from patients on suppressive antiretroviral therapy at concentrations achieved by clinical dosing. PLoS Pathog..

[31] Spivak AM, Planelles V (2016). HIV-1 Eradication: Early Trials (and Tribulations). Trends Mol. Med..

[32] Darcis G, Van Driessche B, Van Lint C (2016). Preclinical shock strategies to reactivate latent HIV-1: an update. Curr. Opin. HIV AIDS.

[33] Williams SA (2004). Prostratin antagonizes HIV latency by activating NF-kappaB. J. Biol. Chem..

[34] Sung TL, Rice AP (2006). Effects of prostratin on Cyclin T1/P-TEFb function and the gene expression profile in primary resting CD4+ T cells. Retrovirology.

[35] Perez M (2010). Bryostatin-1 synergizes with histone deacetylase inhibitors to reactivate HIV-1 from latency. Curr. HIV Res..

[36] Diaz L (2015). Bryostatin activates HIV-1 latent expression in human astrocytes through a PKC and NF-kB-dependent mechanism. Sci. Rep..

[37] Pandelo Jose D (2014). Reactivation of latent HIV-1 by new semi-synthetic ingenol esters. Virology.

[38] Jiang G (2014). Reactivation of HIV latency by a newly modified Ingenol derivative via protein kinase Cdelta-NF-kappaB signaling. AIDS.

[39] Jiang G (2015). Synergistic Reactivation of Latent HIV Expression by Ingenol-3-Angelate, PEP005, Targeted NF-kB Signaling in Combination with JQ1 Induced p-TEFb Activation. PLoS Pathog..

[40] Li Z, Guo J, Wu Y, Zhou Q (2013). The BET bromodomain inhibitor JQ1 activates HIV latency through antagonizing Brd4 inhibition of Tat-transactivation. Nucleic Acids Res..

[41] Lu P (2016). The BET inhibitor OTX015 reactivates latent HIV-1 through P-TEFb. Sci. Rep..

[42] Xing S (2011). Disulfiram reactivates latent HIV-1 in a Bcl-2-transduced primary CD4+ T cell model without inducing global T cell activation. J. Virol..

[43] Doyon G, Zerbato J, Mellors JW, Sluis-Cremer N (2013). Disulfiram reactivates latent HIV-1 expression through depletion of the phosphatase and tensin homolog. AIDS.

[44] Li P (2016). Stimulating the RIG-I pathway to kill cells in the latent HIV reservoir following viral reactivation. Nat. Med..

[45] Winckelmann AA (2013). Administration of a Toll-like receptor 9 agonist decreases the proviral reservoir in virologically suppressed HIV-infected patients. PLoS One.

[46] Novis CL (2013). Reactivation of latent HIV-1 in central memory CD4(+) T cells through TLR-1/2 stimulation. Retrovirology.

[47] Offersen R (2016). A Novel Toll-Like Receptor 9 Agonist, MGN1703, Enhances HIV-1 Transcription and NK Cell-Mediated Inhibition of HIV-1-Infected Autologous CD4+ T Cells. J. Virol..

[48] Bullen CK (2014). New *ex vivo* approaches distinguish effective and ineffective single agents for reversing HIV-1 latency *in vivo*. Nat. Med..

[49] Kimata JT, Rice AP, Wang J (2016). Challenges and strategies for the eradication of the HIV reservoir. Curr. Opin. Immunol..

[50] Tu Y (2011). The discovery of artemisinin (qinghaosu) and gifts from Chinese medicine. Nat. Med..

[51] Hori T (2015). Procyanidin trimer C1 derived from Theobroma cacao reactivates latent human immunodeficiency virus type 1 provirus. Biochem. Biophys. Res. Commun..

[52] Wang C (2015). A Natural Product from Polygonum cuspidatum Sieb. Et Zucc. Promotes Tat-Dependent HIV Latency Reversal through Triggering P-TEFb’s Release from 7SK snRNP. PLoS One.

[53] Yan X (2014). Processing of kansui roots stir-baked with vinegar reduces kansui-induced hepatocyte cytotoxicity by decreasing the contents of toxic terpenoids and regulating the cell apoptosis pathway. Molecules.

[54] Cary DC, Fujinaga K, Peterlin BM (2016). Euphorbia Kansui Reactivates Latent HIV. PLoS One.

[55] Hou JJ (2014). A single, multi-faceted, enhanced strategy to quantify the chromatographically diverse constituents in the roots of Euphorbia kansui. J. Pharm. Biomed. Anal..

[56] Ding D (2013). Involvement of histone methyltransferase GLP in HIV-1 latency through catalysis of H3K9 dimethylation. Virology.

[57] Qu X (2013). Zinc-finger-nucleases mediate specific and efficient excision of HIV-1 proviral DNA from infected and latently infected human T cells. Nucleic Acids Res..

[58] Jiang G, Dandekar S (2015). Targeting NF-kappaB signaling with protein kinase C agonists as an emerging strategy for combating HIV latency. AIDS Res. Hum. Retroviruses.

[59] Kulkosky J (2001). Prostratin: activation of latent HIV-1 expression suggests a potential inductive adjuvant therapy for HAART. Blood.

[60] Jordan A, Bisgrove D, Verdin E (2003). HIV reproducibly establishes a latent infection after acute infection of T cells *in vitro*. EMBO J..

[61] Laird GM (2015). *Ex vivo* analysis identifies effective HIV-1 latency-reversing drug combinations. J. Clin. Investig..

[62] Darcis G (2015). An In-Depth Comparison of Latency-Reversing Agent Combinations in Various *In Vitro* and *Ex Vivo* HIV-1 Latency Models Identified Bryostatin-1 + JQ1 and Ingenol-B + JQ1 to Potently Reactivate Viral Gene Expression. PLoS Pathog..

[63] Fenaux P (2005). Inhibitors of DNA methylation: beyond myelodysplastic syndromes. Nat. Clin. Pract. Oncol..

[64] Boehm D (2013). BET bromodomain-targeting compounds reactivate HIV from latency via a Tat-independent mechanism. Cell Cycle.

[65] Blankson JN, Persaud D, Siliciano RF (2002). The challenge of viral reservoirs in HIV-1 infection. Annu. Rev. Med..

[66] Shan L (2013). A novel PCR assay for quantification of HIV-1 RNA. J. Virol..

[67] Gschwendt M (1996). Inhibition of protein kinase C mu by various inhibitors. Differentiation from protein kinase c isoenzymes. FEBS Lett..

[68] Martiny-Baron G (1993). Selective inhibition of protein kinase C isozymes by the indolocarbazole Go 6976. J. Biol. Chem..

[69] Cole DC (2008). Identification, characterization and initial hit-to-lead optimization of a series of 4-arylamino-3-pyridinecarbonitrile as protein kinase C theta (PKCtheta) inhibitors. J. Med. Chem..

[70] Bedoya LM (2009). SJ23B, a jatrophane diterpene activates classical PKCs and displays strong activity against HIV *in vitro*. Biochem. Pharmacol..

[71] Oeckinghaus A, Ghosh S (2009). The NF-kappaB family of transcription factors and its regulation. Cold Spring Harb. Perspect. Biol..

[72] Mbonye UR (2013). Phosphorylation of CDK9 at Ser175 enhances HIV transcription and is a marker of activated P-TEFb in CD4(+) T lymphocytes. PLoS Pathog..

[73] Rice AP (2016). Cyclin-dependent kinases as therapeutic targets for HIV-1 infection. Expert Opin. Ther. Targets.

[74] Wang HY (2012). Bioactivity-guided isolation of antiproliferative diterpenoids from Euphorbia kansui. Phytother. Res..

[75] Zheng WF, Cui Z, Zhu Q (1998). Cytotoxicity and antiviral activity of the compounds from Euphorbia kansui. Planta Med..

[76] Zeng Y (1994). Screening of Epstein-Barr virus early antigen expression inducers from Chinese medicinal herbs and plants. Biomed. Environ. Sci..

[77] Nunomura S, Kitanaka S, Ra C (2006). 3-O-(2,3-dimethylbutanoyl)-13-O-decanoylingenol from Euphorbia kansui suppresses IgE-mediated mast cell activation. Biol. Pharm. Bull..

[78] Khiev P (2012). Ingenane-type diterpenes with a modulatory effect on IFN-gamma production from the roots of Euphorbia kansui. Arch. Pharm. Res..

[79] Wu TS (1991). Antitumor agents, 119. Kansuiphorins A and B, two novel antileukemic diterpene esters from Euphorbia kansui. J. Nat. Prod..

[80] Wang LY (2003). Euphane and tirucallane triterpenes from the roots of Euphorbia kansui and their *in vitro* effects on the cell division of Xenopus. J. Nat. Prod..

[81] Doan HQ, Gulati N, Levis WR (2012). Ingenol mebutate: potential for further development of cancer immunotherapy. J. Drugs Dermatol..

[82] Aditya S, Gupta S (2013). Ingenol mebutate: A novel topical drug for actinic keratosis. *Indian Dermatol*. Online J..

[83] Fujiwara M (1996). Mechanism of selective inhibition of human immunodeficiency virus by ingenol triacetate. Antimicrob. Agents Chemother..

[84] Fujiwara M (1998). Upregulation of HIV-1 replication in chronically infected cells by ingenol derivatives. Arch. Virol..

[85] Warrilow D (2006). HIV type 1 inhibition by protein kinase C modulatory compounds. AIDS Res. Hum. Retroviruses.

[86] Abreu CM (2014). Dual role of novel ingenol derivatives from Euphorbia tirucalli in HIV replication: inhibition of de novo infection and activation of viral LTR. PLoS One.

[87] Wender PA (1986). Analysis of the phorbol ester pharmacophore on protein kinase C as a guide to the rational design of new classes of analogs. Proc. Natl. Acad. Sci. USA.

[88] Newton AC (1995). Protein kinase C: structure, function, and regulation. J. Biol. Chem..

[89] Kazi JU, Kabir NN, Ronnstrand L (2013). Protein kinase C (PKC) as a drug target in chronic lymphocytic leukemia. Med. Oncol..

[90] Wu-Zhang AX, Newton AC (2013). Protein kinase C pharmacology: refining the toolbox. Biochem. J..

[91] Soh JW, Weinstein IB (2003). Roles of specific isoforms of protein kinase C in the transcriptional control of cyclin D1 and related genes. J. Biol. Chem..

[92] Zhong H, May MJ, Jimi E, Ghosh S (2002). The phosphorylation status of nuclear NF-kappa B determines its association with CBP/p300 or HDAC-1. Mol. Cell.

[93] Wei P (1998). A novel CDK9-associated C-type cyclin interacts directly with HIV-1 Tat and mediates its high-affinity, loop-specific binding to TAR RNA. Cell.

[94] Kim YK (2002). Phosphorylation of the RNA polymerase II carboxyl-terminal domain by CDK9 is directly responsible for human immunodeficiency virus type 1 Tat-activated transcriptional elongation. Mol. Cell. Biol..

[95] Zhou Q, Li T, Price DH (2012). RNA polymerase II elongation control. Annu. Rev. Biochem..

[96] Sgarbanti M, Battistini A (2013). Therapeutics for HIV-1 reactivation from latency. Curr. Opin. Virol..

[97] Spivak AM (2016). Janus kinase inhibition suppresses PKC-induced cytokine release without affecting HIV-1 latency reversal *ex vivo*. Retrovirology.

[98] Martin AR (2017). Rapamycin-mediated mTOR inhibition uncouples HIV-1 latency reversal from cytokine-associated toxicity. J. Clin. Invest..

[99] Ying H (2012). Selective histonedeacetylase inhibitor M344 intervenes in HIV-1 latency through increasing histone acetylation and activation of NF-kappaB. PLoS One.

[100] Wang P (2013). As2O3 synergistically reactivate latent HIV-1 by induction of NF-kappaB. Antiviral Res..

[101] Wang P (2014). Specific reactivation of latent HIV-1 with designer zinc-finger transcription factors targeting the HIV-1 5′-LTR promoter. Gene Ther..

