# Supplementary material for: Reactivation of HIV-1 from Latency by an Ingenol Derivative from Euphorbia Kansui
Source: Sci Rep. 2017 Aug 25;7:9451. doi: 10.1038/s41598-017-07157-0 (PMC5573388; doi:10.1038/s41598-017-07157-0)
Supplement: Supplementary file 1 — Supplementary Information [file 41598_2017_7157_MOESM1_ESM.doc]

***Supplementary Data for***

**Reactivation of HIV-1 From Latency by an Ingenol Derivative from** ***Euphorbia* *Kansui***

**Pengfei Wang**^1^**, Panpan Lu**^1^**, Xiying Qu**^1^**, Yinzhong Shen**^2^**, Hanxian Zeng**^1^**, Xiaoli Zhu**^1^**, Yuqi Zhu**^1^**, Xian Li**^1^**, Hao Wu**^3^**, Jianqing Xu**^2^**, Hongzhou Lu**^2^**, Zhongjun Ma**^4*^**, Huanzhang Zhu**^1*^

^1^State Key Laboratory of Genetic Engineering and Key Laboratory of Medical Molecular Virology of Ministry of Education/Health, Institute of Genetics, School of Life Sciences, Fudan University, Shanghai 200438, China.

^2^Department of Infectious Diseases, and Key Laboratory of Medical Molecular Virology of Ministry of Education/Health, Shanghai Public Health Clinical Center, Fudan University, Shanghai 200433, China.

^3^Center for Infectious Diseases, Beijing You'an Hospital, Capital Medical University, Beijing 100069, China.

^4^Institute of Marine Biology, Ocean College, Zhejiang University, Hangzhou 310058, China.

^*^Correspondence and requests for materials should be addressed to Huanzhang Zhu [(email: hzzhu@fudan.edu.cn)](mailto:(email: hzzhu@fudan.edu.cn)) or Zhongjun Ma (email: [mazj@zju.edu.cn](mailto:mazj@zju.edu.cn))

**Supplementary Figure 1. Reactivation of latent HIV-1 by effective fractions from *Euphorbia kansui*.** (**a**) C11 cells were treated with methylene chloride (EF-MC) or petroleum ether effective fractions (EF-PE) from *Euphorbia kansui*, both at 1 μg/ml concentration for 48 h, and the percentage of GFP-positive cells was measured by flow cytometry. (**b**) Dose-dependent and (**c**) time-dependent curves of EF-MC and EF-PE on HIV-1 production in C11 cells are shown.

**Supplementary Figure 2. Effects of EK-16A on cell viability.** C11 cells (**a**) and J-Lat 10.6 cells (**b**) were treated with EK-16A at the indicated concentration for 72 h and then cell viability were measured by CCK-8 kit (Dojindo). The division of OD450 between treated and control groups indicate the percentage of cell viability. Data show the means ± standard deviations in three independent experiments.

**Supplementary Figure 3. EK-16A reactivates HIV-1 from infected resting CD4^+^ T cells *ex vivo*.** Resting CD4 T cells isolated from two cART-suppressed HIV-1-infected patients were treated with either EK-16A (0.05 μM) or PHA (5 μg/ml) for 18 h. Supernatant HIV-1 mRNA levels were detected by qRT-PCR and presented as fold induction relative to mock-treated control.

**Supplementary Figure 4. Plasmid map of shRNA-expressing vector.** The shRNAs targeting PKCγ gene were cloned into the *Bbs*I and *Bam*HI sites.


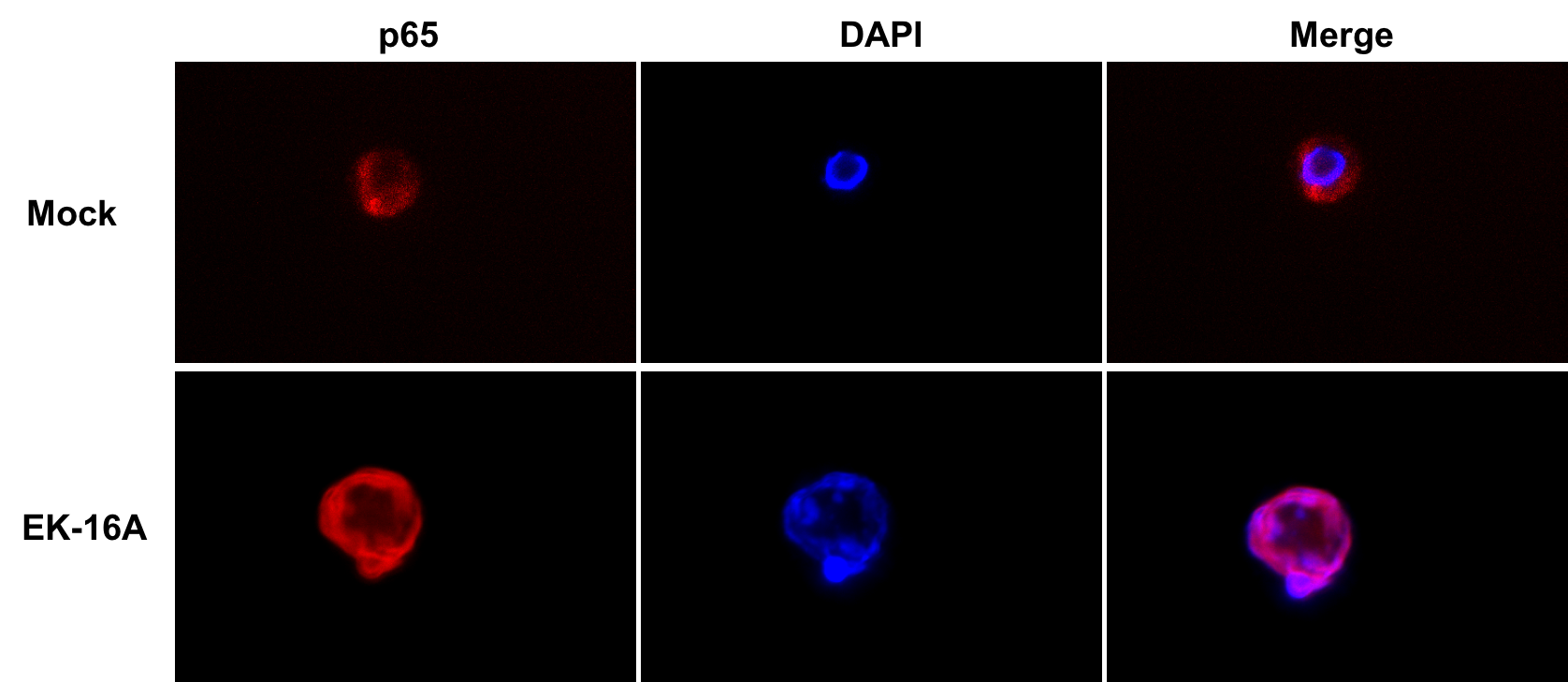


**Supplementary Figure 5. Subcellular localization of p65.** Immunofluorescence analysis of the p65 protein in C11 cells mock treated or treated with 0.05 μM EK-16A for 30 min. Subcellular localization of p65 was determined via indirect immunofluorescence employing rabbit polyclonal anti-p65 and goat anti-rabbit antibody coupled to Alexa-555. DAPI staining was used to determine the region of nuclei and to assess gross cell morphology.

**Supplementary Figure 6. EK-16A promotes the up-regulation of Cyclin T1 RNA.** C11 cells were stimulated with prostratin (1 μM) for 30 min or ΕΚ-16Α (0.05 μM) for 2 h, and RT-PCR was performed to detect the Cyclin T1 mRNA expression levels. Human *β-globin* gene was used as internal control for RNA input. Data show the means ± standard deviations in four independent experiments.
